# Parvovirus B19 Infection as a Potential Trigger for Systemic Diseases

**DOI:** 10.1002/ccr3.71148

**Published:** 2025-10-05

**Authors:** Giulia Ciccarese, Serena Varesano, Gaetano Serviddio, Francesco Drago

**Affiliations:** ^1^ Section of Dermatology, Department of Medical and Surgical Sciences University of Foggia Foggia Italy; ^2^ Hygiene Unit IRCCS Ospedale Policlinico San Martino Genoa Italy; ^3^ Internal Medicine, Liver Unit, C.U.R.E. (University Centre for Liver Disease Research and Treatment), Department of Medical and Surgical Sciences University of Foggia Foggia Italy; ^4^ Dermatology Clinic Villa Montallegro Health Clinic Genoa Italy

**Keywords:** dermatology, global health, infectious diseases, virology

## Abstract

It is crucial to perform direct methods like polymerase chain reaction (PCR) beyond serology to diagnose an active viral infection that can trigger a cutaneous/systemic disease, such as fibromyalgia. Indeed, measuring the viral load in tissues, plasma, and serum are diagnostic tools that permit the follow‐up of an active infection.

Dear Editor,

The interesting article by A. Nigro about the onset of fibromyalgia following Parvovirus B19 infection recently published in your journal [1] prompted us to describe our experience. Fibromyalgia is a chronic pain disorder with unclear etiology, which negatively impacts the quality of life [2]. We agree with the author that Parvovirus B19 and human T‐lymphotropic virus type 1 (HTLV‐1) infections may trigger fibromyalgia as well as physical trauma and emotional stress [1, 2]. Many studies suggested that numerous viral and bacterial infectious agents, such as hepatitis B and C viruses, human immunodeficiency virus (HIV), Epstein Barr virus (EBV), severe acute respiratory syndrome coronavirus 2 (SARS‐CoV‐2) [3], *Borrelia burgdorferi* , *Helicobacter pylori* , and also some vaccinations, may be associated with the development of fibromyalgia [2, 3]. Furthermore, the role of other viruses potentially pathogenic to the nervous system, namely human herpesvirus (HHV)‐6 and HHV‐7, has been investigated [4]. Authors detected a higher frequency of active HHV‐6 and HHV‐7 infection in fibromyalgia patients than in control individuals [4]; moreover, a statistically significant correlation between the dysfunction of small fibers with changes in warm and cold sensations and an active HHV‐6 infection was found [4]. These findings were confirmed by the reduction of dermal unmyelinated nerve fiber bundles in skin samples of FM patients, compared with the healthy controls; this reduction resulted in impaired small fiber function causing increased thresholds of cold and warm detection [5].

HHV‐6 and HHV‐7 are ubiquitous viruses, usually acquired through saliva droplets in childhood. The primary infection causes exanthema subitum, the most common exanthematic fever in children under 3 years [6]. After the primary infection, these viruses remain latent in the body, mainly associated with peripheral blood mononuclear cells (PBMCs) and salivary glands, and can reactivate during states of immunosuppression. The HHV‐6 and HHV‐7 reactivation has a causative role in the pathogenesis of pityriasis rosea, an acute exanthematous disease commonly affecting children and adults between 10 and 35 years of age [6, 7]. The detection of HHV‐6 and HHV‐7 plasma DNA (a marker of active viral infection) was revealed only in fibromyalgia patients and not in healthy controls, suggesting these viruses' potential role in triggering the disease's onset or reactivation [4]. The mechanism through which an infectious viral/bacterial agent may cause fibromyalgia is not completely clear. Recent blood transcriptome analysis and machine learning studies shed new light on the crosstalk between SARS‐CoV‐2 infection and fibromyalgia; this crosstalk may represent a model through which other pathogens may trigger the onset of inflammatory diseases like fibromyalgia. Indeed, SARS‐CoV‐2 infection may activate several fibromyalgia‐related genes, which are responsible for synthesizing and regulating various cytokines that represent key mediators of pain in fibromyalgia [8]. Indeed, fibromyalgia has been associated with many cytokine abnormalities including increased levels of IL‐2, IL‐8, IL‐1, and IL‐6 [8]. More specifically, IL‐1 and IL‐6 are known as “cellular cytokines” which drive, albeit not exclusively, cytotoxic and antiviral response [9].

As previously reported for HHV‐6/7, serologic assays alone cannot be sufficient to diagnose viral reactivations, whereas direct methods such as polymerase chain reaction (PCR) are preferred. In particular, bona fide quantitative methods, which measure the HHV‐6 and HHV‐7 viral load in tissues, blood cells, and, particularly, in plasma and serum, are diagnostic and permit the follow‐up of active infections [10, 11, 12]. Likewise, especially in adults, to confirm a Parvovirus B19 active infection in fibromyalgia and other clinical conditions, in addition to the findings of serology, the viral DNA should be detected in serum samples. In a previous retrospective study on 390 patients with atypical exanthems, including 120 children and 270 adults, we performed serology for specific Parvovirus B19 IgG and IgM antibodies in the acute and convalescent phases as well as PCR for detection of Parvovirus B19 DNA in serum [13]. Atypical exanthems related to Parvovirus B19 infection were found in 5% of the pediatric and 5.2% of the adult patients [13]. Regarding serology, all children showed, in the acute phase of the exanthem, Parvovirus B19 IgM antibodies and low/absent levels of IgG antibodies; in the convalescent phase (3 weeks after the onset of the rash), IgM antibodies were still detectable and IgG antibody levels were increased, indicating a primary infection by Parvovirus B19 in children. Among the adults in the acute phase of the exanthem, the specific Parvovirus B19 IgM and IgG antibodies were detected in all patients (100%); in addition, 11 patients (79%) had also a positive PCR test. In the convalescent phase, only seven patients still presented Parvovirus B19 IgM antibodies, whereas the Parvovirus DNA was always negative. These data suggest a virus reactivation in adults [13].

Unfortunately, Nigro did not describe the pattern of the exanthems related to Parvovirus B19 infection in the patient with fibromyalgia. In our series, we found that in the adult patients the eruption mostly consisted of purpuric lesions, in line with the literature [14, 15, 16]. Moreover, most adult patients had maculo‐papular lesions on the trunk, neck, and extensor surfaces of the limbs with a central fading that sometimes gave a reticulated appearance. In children, the exanthem was characterized by the typical slapped cheek sign as the initial manifestation followed by macule, papules, and petechiae on the trunk and limbs. Palms and soles were always spared [6, 13]. Enanthem with a maculo‐papular and petechial pattern was often observed both in adults and children [6, 17]. In conclusion, the pathogenic bases of the link between infections and fibromyalgia are still to be fully elucidated. To diagnose an active viral infection that can potentially trigger a cutaneous or systemic disease such as fibromyalgia, it is crucial to perform direct methods like PCR beyond serology. More specifically, bona fide quantitative methods, which measure the viral load in tissues, blood cells, and, particularly, in plasma and serum, are diagnostic tools also permitting the follow‐up of an active infection [12].

## Author Contributions

**Giulia Ciccarese:** conceptualization, data curation, formal analysis, investigation, methodology, project administration, resources, software, supervision, validation, visualization, writing – original draft, writing – review and editing. **Serena Varesano:** formal analysis, writing – review and editing. **Gaetano Serviddio:** writing – review and editing. **Francesco Drago:** conceptualization, data curation, methodology, supervision, validation, writing – review and editing.

## Ethics Statement

The authors have nothing to report.

## Consent

The authors have nothing to report.

## Conflicts of Interest

The authors declare no conflicts of interest.

## Linked Articles

Onset of fibromyalgia symptoms following parvovirus b19 infection: A case report and review of the literature, https://doi.org/10.1002/ccr3.70188.

## Data Availability

The authors declare that the data supporting the findings of this study is available upon reasonable request.
